# Metabolic and Laboratory Biomarkers in Early-Onset Versus Late-Onset Colorectal Cancer: A Case–Control Study

**DOI:** 10.3390/cancers18132152

**Published:** 2026-07-03

**Authors:** Mohamed H. Eldesouki, Ahmed E. Salem, Youssef Hafez, Ezz ElDien A. Ibrahim, Mohammed Y Youssef, Fatima Khan, Mohammed Alomari, Sherif E. ElHananfi, Aasma Shaukat

**Affiliations:** 1 Department of Internal Medicine, New York Medical College at Saint Michael’s Medical Center, Newark, NJ 07102, USA; 2 Division of Gastroenterology and Hepatology, Cleveland Clinic Florida, Weston, FL 33331, USA; ahmed.e.salem@hotmail.com; 3 Division of Gastroenterology and Hepatology, School of Medicine, St. Louis University, Saint Louis, MO 63104, USA; youssef.hafez@health.slu.edu; 4 Department of Internal Medicine, University of Missouri-Columbia, Columbia, MO 65212, USA; ezzeldienibrahimm@gmail.com; 5 Department of Internal Medicine, Hunt Regional Medical Center, Greenville, TX 75401, USA; mohammedyoussef96@yahoo.com; 6 Department of Internal Medicine, Maimonides Medical Center, State University New York Downstate, Brooklyn, NY 11219, USA; fatkhan@maimo.org; 7 Division of Gastroenterology and Hepatology, University of Arkansas for Medical Sciences, Little Rock, AR 72205, USA; malomari@uams.edu; 8 Division of Gastroenterology, Tyler School of Medicine, University of Texas, Tyler, TX 75708, USA; sherif.elhanafi@uttyler.edu; 9 Division of Gastroenterology and Hepatology, Department of Medicine, NYU Grossman School of Medicine, New York, NY 10016, USA; aasma.shaukat@nyulangone.org

**Keywords:** early-onset colorectal cancer, late-onset colorectal cancer, colorectal cancer, metabolic syndrome, obesity, MASLD

## Abstract

**Background:** The incidence of early-onset colorectal cancer (EOCRC) is rising, yet the relative contribution of metabolic, inflammatory, and laboratory abnormalities remains incompletely defined. Objectives: We compared these associations between EOCRC and late-onset colorectal cancer (LOCRC) while addressing the possibility that some laboratory abnormalities may reflect occult cancer rather than antecedent risk. **Methods:** We conducted a matched case–control study using the TriNetX US Network. Adults diagnosed with CRC between 2010 and 2023 were identified as EOCRC (18–49 years) or LOCRC (50–75 years). Patients with prior malignancy, inflammatory bowel disease, hereditary or familial CRC risk, or prior colectomy were excluded. Three separate analyses were performed. First, a direct EOCRC-versus-LOCRC comparison evaluated gastrointestinal symptoms during the 6 months preceding diagnosis. Second, EOCRC and LOCRC were each compared with their respective matched cancer-free controls to assess clinical, metabolic, and laboratory features during the 24 months preceding diagnosis. When multiple laboratory values were available, the most recent value preceding the index date was used. Conditional logistic regression estimated adjusted odds ratios with 95% confidence intervals, with Bonferroni correction applied for multiple comparisons. **Results:** The direct matched EOCRC-versus-LOCRC comparison included 7752 patients with CRC, comprising 2584 with EOCRC and 5168 with LOCRC. EOCRC more frequently presented with rectal bleeding, abdominal pain, diarrhea, iron-deficiency anemia, and weight loss. Rectal tumors were more common in EOCRC, whereas proximal tumors were more common in LOCRC. In separate control-based analyses, 3217 patients with EOCRC and 12,112 patients with LOCRC were compared with 6434 and 24,336 matched cancer-free controls, respectively. The strongest independent features associated with EOCRC were severe obesity (aOR 2.61), microcytosis (aOR 2.29), low ferritin (aOR 2.11), and elevated C-reactive protein (aOR 1.87). Similar but generally attenuated associations were observed in LOCRC. In adjusted EOCRC-versus-LOCRC analyses, obesity (aOR 1.38), metabolic syndrome (aOR 1.41), and MASH (aOR 1.22) remained more closely associated with EOCRC. **Conclusions:** EOCRC is associated with a distinct clinical–metabolic phenotype, with more pronounced metabolic, inflammatory, and hematologic abnormalities than LOCRC. These findings should be interpreted as hypothesis-generating prediagnostic associations, not as validated predictors or causal risk factors.

## Simple Summary

Early-onset colorectal cancer (EOCRC) is increasing, but the clinical and biological factors that distinguish it from late-onset colorectal cancer (LOCRC) remain incompletely understood. In this matched case–control study, we compared symptoms, tumor location, and prediagnostic metabolic and laboratory features between EOCRC and LOCRC. EOCRC patients more often presented with rectal bleeding, abdominal pain, diarrhea, iron-deficiency anemia, and weight loss, and were more likely to have rectal tumors. Compared with matched cancer-free controls, EOCRC showed stronger associations with obesity, metabolic syndrome, microcytosis, low ferritin, and elevated C-reactive protein than LOCRC. These findings support EOCRC as a distinct clinical–metabolic phenotype. Because laboratory abnormalities were measured within the two years before diagnosis, they should be interpreted as prediagnostic correlates that may partly reflect occult malignancy rather than proven causal risk factors. Prospective and external validation studies are needed before these markers can be used for screening or EHR-based risk stratification.

## 1. Introduction

Cancer development is a multistep process shaped by inherited susceptibility, somatic genomic alterations, epigenetic dysregulation, immune escape, inflammation, and interactions between host metabolism and the tumor microenvironment. Contemporary cancer treatment has also evolved from surgery, cytotoxic chemotherapy, and radiotherapy to include targeted therapy, immunotherapy, cellular therapy, and molecularly guided approaches, emphasizing the importance of linking clinical phenotypes with underlying biology [1]. Colorectal cancer (CRC) remains the third most commonly diagnosed malignancy and the second leading cause of cancer death worldwide [2,3,4]. Screening initiatives have successfully decreased the incidence and mortality of CRC in older adults over the last thirty years [5]. However, a concerning countertrend has surfaced. Since the mid-1990s, early-onset CRC (EOCRC), defined as diagnosis before the age of 50, has increased steadily at a rate of approximately 2% annually and now accounts for 10 to 14% of all CRC diagnoses [6,7]. By 2022, CRC had become the leading cause of cancer death among American men under 50 and the second leading cause among women in this age group [7]. Interestingly, there is a birth cohort pattern to this increase, with those born after 1960 having increasingly higher risks; those born in the 1990s are at least four times more at risk than those born three decades earlier [8]. The etiology of this generational shift remains incompletely understood. While hereditary cancer syndromes explain a larger fraction of EOCRC than late-onset disease, most cases are sporadic, with no identifiable genetic predisposition [6]. Attention has therefore turned to modifiable risk factors, particularly metabolic dysfunction. The dramatic rise in childhood and adult obesity, metabolic syndrome, type 2 diabetes, and metabolic-associated steatotic liver disease (MASLD) over the same period is unlikely to be coincidental [7,9,10]. For instance, obesity alone confers about an 88% increased risk of EOCRC, and metabolic syndrome raises risk by 20% to 50% [7,11,12,13].

Inflammation also plays a key role in colorectal carcinogenesis. Inflammatory bowel disease (IBD) is nearly three times more prevalent among EOCRC patients compared with those diagnosed after age 50 [7,14]. Beyond clinical risk factors, routine laboratory abnormalities, including iron deficiency anemia, elevated inflammatory markers, and dyslipidemia, have emerged as potential early signals of colorectal neoplasia, yet their role as independent features of EOCRC has not been systematically evaluated at population scale [15,16,17]. Recognizing these trends, major guidelines have responded accordingly. The US Preventive Services Task Force lowered the recommended screening age from 50 to 45 in 2021, a recommendation also endorsed by the American Cancer Society and the American College of Gastroenterology [18,19,20].

Despite growing awareness, the factors driving EOCRC remain incompletely defined. Prior studies have often evaluated metabolic, inflammatory, and laboratory markers in isolation, and few have directly compared their relative associations in EOCRC versus late-onset CRC (LOCRC). To address this gap, we conducted a large, matched case–control study using the TriNetX US Collaborative Network. We aimed to identify prediagnostic clinical and laboratory features associated with EOCRC and to determine whether these factors differ meaningfully from those observed in LOCRC.

## 2. Materials and Methods

### 2.1. Data Source and Design

We conducted a multicenter matched case–control study using the TriNetX U.S. Network (https://trinetx.com; accessed 18 April 2025), a federated electronic health record database comprising de-identified data from 67 health care organizations across the United States [21]. Participating organizations include academic medical centers, affiliated hospitals, satellite facilities, and outpatient clinics. The database captures patient-level information on diagnoses, procedures, medications, laboratory measurements, and clinical encounters. Diagnoses were identified using International Classification of Diseases, Ninth and Tenth Revision, Clinical Modification codes; procedures were identified using ICD-10-PCS and Current Procedural Terminology codes; laboratory measurements were identified using Logical Observation Identifiers Names and Codes; medications were identified using RxNorm and Veterans Affairs codes; and physical measurements were identified using TriNetX-specific clinical observation codes. For encounters occurring before 2015, ICD-9-CM codes were mapped to corresponding ICD-10-CM codes using TriNetX General Equivalence Mappings. Because the TriNetX database contains only de-identified patient information, this study was exempt from institutional review board approval and from the requirement for informed consent; no consent form was administered, as the work was a secondary analysis of existing data involving no intervention or interaction with human subjects [22]. De-identification is performed at the network level in accordance with the de-identification standard defined in Section §164.514(a) of the Health Insurance Portability and Accountability Act (HIPAA) Privacy Rule and is attested through a formal determination by a qualified expert as defined in Section §164.514(b)(1) of the Privacy Rule [22]. To preserve patient confidentiality, TriNetX suppresses patient counts fewer than 10.

### 2.2. Study Population

Adults with the first recorded diagnosis of CRC between January 2010 and December 2023 were identified. The date of the first qualifying CRC diagnosis was defined as the index date. Patients were excluded if they had any prior malignancy, inflammatory bowel disease, hereditary colorectal cancer syndrome, including Lynch syndrome or familial adenomatous polyposis, family history of gastrointestinal (GI) malignancy, or colectomy before the index date. These exclusions were applied before cohort construction, matching, and model development to reduce confounding from established hereditary, inflammatory, or treatment-related CRC risk factors. Two age-defined CRC cohorts were constructed. EOCRC was defined as CRC diagnosed between ages 18 and 49 years, and LOCRC was defined as CRC diagnosed between ages 50 and 75 years. Cancer-free controls were selected from adults in the TriNetX network with at least one documented healthcare encounter during the study period and no recorded diagnosis of colorectal or any other malignancy at any time. The same exclusion criteria applied to cases, inflammatory bowel disease, hereditary colorectal cancer syndrome, family history of GI malignancy, and prior colectomy, were also applied to controls. Because controls had no diagnosis date to serve as a natural index date, each control was assigned an index date corresponding to a qualifying healthcare encounter occurring within the same calendar period as that of the matched case. All ascertainment windows for controls—the 6-month window for GI symptoms and the 24-month window for clinical, metabolic, and laboratory variables—were anchored to this assigned index date, so that exposures were assessed over time intervals equivalent to those used for cases.

Three matched analytic comparisons were then performed. First, patients with EOCRC were directly compared with patients with LOCRC to compare clinical presentation, tumor location, and baseline metabolic and laboratory features according to age at CRC diagnosis. GI symptoms were assessed during the 6 months preceding the index date to capture the clinical presentation most proximal to diagnosis. We then performed two parallel control-based analyses to identify prediagnostic clinical, metabolic, and laboratory features associated with both EOCRC and LOCRC. First, EOCRC cases were compared with matched cancer-free controls, with each EOCRC case matched to up to two controls. Second, LOCRC cases were compared with matched cancer-free controls, with each LOCRC case matched to up to two controls. These control-based analyses used separate age-anchored matched sets and therefore were not expected to have the same denominator as the direct EOCRC-versus-LOCRC comparison. All codes utilized in this study are listed in Supplementary Table S1 and S2.

### 2.3. Covariates and Variable Ascertainment

GI symptoms reflecting clinical presentation were ascertained during the 6-month period preceding the index date. These included rectal bleeding or hematochezia, abdominal pain, change in bowel habits, diarrhea, constipation, iron-deficiency anemia, unintentional weight loss, bowel obstruction symptoms, nausea or vomiting, and tenesmus. Clinical, metabolic, and laboratory variables were ascertained during the 24-month period preceding the index date to evaluate prediagnostic risk profiles. Clinical variables included obesity, body mass index (BMI), type 2 diabetes mellitus, metabolic syndrome, metabolic dysfunction-associated steatotic liver disease/MASLD, smoking, physical inactivity, alcohol use, and prior colonoscopy or CRC screening. Laboratory variables included hemoglobin, mean corpuscular volume (MCV), red cell distribution width (RDW), platelet count, hemoglobin A1c, lipid profile components, inflammatory markers, ferritin, and transferrin saturation.

Variables were categorized using clinically relevant thresholds defined a priori, including anemia, microcytosis, thrombocytosis, elevated hemoglobin A1c, low high-density lipoprotein cholesterol, elevated low-density lipoprotein cholesterol, hypertriglyceridemia, elevated C-reactive protein, elevated erythrocyte sedimentation rate, low ferritin, and low transferrin saturation. The 24-month prediagnostic window was selected a priori to balance two competing goals: capturing clinically meaningful abnormalities documented before CRC diagnosis while limiting exposure misclassification from remote measurements less likely to represent the clinical state immediately preceding diagnosis. When multiple laboratory values were available during the 24-month ascertainment window, the value closest to but preceding the index date was used, rather than an averaged value or the single most extreme measurement. Because occult CRC may already be present during this interval, these variables were treated as prediagnostic correlates rather than definitive antecedent risk factors.

### 2.4. Statistical Analysis

Baseline characteristics were summarized using means with standard deviations for continuous variables and counts with percentages for categorical variables. Between-group comparisons were performed within the matched analytic cohorts. All analyses were conducted using R (R Foundation for Statistical Computing) within the TriNetX analytics platform. The primary analytic workflow began with the direct EOCRC versus LOCRC comparison, followed by separate EOCRC-versus-control and LOCRC-versus-control analyses. Matching was performed separately within each analytic comparison before regression modeling. Propensity scores were estimated by multivariable logistic regression on the specified matching covariates, and cases were matched to comparators using greedy nearest-neighbor matching without replacement. For the direct EOCRC versus LOCRC comparison, patients were matched on sex, race, ethnicity, prior healthcare utilization (defined as the number of prior hospitalizations and outpatient clinic visits), and prior colonoscopy. Age was not included as a matching variable in this comparison because age defined the EOCRC and LOCRC cohorts. For the EOCRC-versus-control and LOCRC-versus-control analyses, cases were matched to cancer-free controls on age at index date, sex, race, ethnicity, calendar year of index date, prior healthcare utilization, and prior colonoscopy history. Metabolic and laboratory variables were not included in the matching model because these variables were evaluated as primary variables of interest. Conditional logistic regression was used for all matched comparisons to account for the matched study design. Associations between clinical and laboratory variables and CRC status were reported as adjusted odds ratios (aORs) with 95% confidence intervals (CIs). Missing laboratory data were handled using multiple imputation by chained equations under a missing-at-random assumption, generating 20 imputed datasets. The imputation model incorporated the matching covariates together with the clinical, metabolic, and laboratory variables. Imputed distributions were reviewed for clinical plausibility, and estimates were pooled across imputed datasets using Rubin’s rules. Firth penalized regression was applied when sparse data or quasi-complete separation was encountered. To account for multiple comparisons, Bonferroni correction was selected as a conservative family-wise error approach because the analysis evaluated multiple correlated biomarkers and symptoms in a hypothesis-generating setting. For the EOCRC versus LOCRC GI symptom analysis, statistical significance was defined as *p* < 0.005, corresponding to 10 symptom-level comparisons. For the metabolic and laboratory comparisons of EOCRC and LOCRC with their respective matched controls, statistical significance was defined as *p* < 0.0014, corresponding to 36 comparisons. Unadjusted *p*-values are reported in the tables, and statistical significance was interpreted according to the Bonferroni-corrected thresholds. Formal prediction-model development, calibration assessment, decision-curve analysis, external validation, sex-specific modeling, race/ethnicity-stratified modeling, stage-adjusted analysis, molecular subtype analysis, and alternative exposure-window sensitivity analyses were not performed in the present TriNetX analysis and are acknowledged as limitations and priorities for future work. All tests were two-sided.

## 3. Results

### 3.1. Direct Comparison of Matched EOCRC and LOCRC Cohorts

#### 3.1.1. Baseline Characteristics Between EOCRC and LOCRC Cohorts

A total of 7752 patients with CRC were included, comprising 2584 EOCRC and 5168 LOCRC cases. Compared with LOCRC, EOCRC included a higher proportion of Black (16.6% vs. 12.9%) and Hispanic patients (16.0% vs. 13.4%), whereas White patients were more common in LOCRC (63.3% vs. 58.4%). EOCRC patients were more likely to have obesity (48.0% vs. 40.0%), current smoking (27.6% vs. 22.7%), metabolic syndrome (21.5% vs. 16.5%), and MASLD (13.7% vs. 11.5%) (Table 1).

#### 3.1.2. Differences in Prediagnostic Gastrointestinal Symptoms Between EOCRC and LOCRC

**Figure 1:**
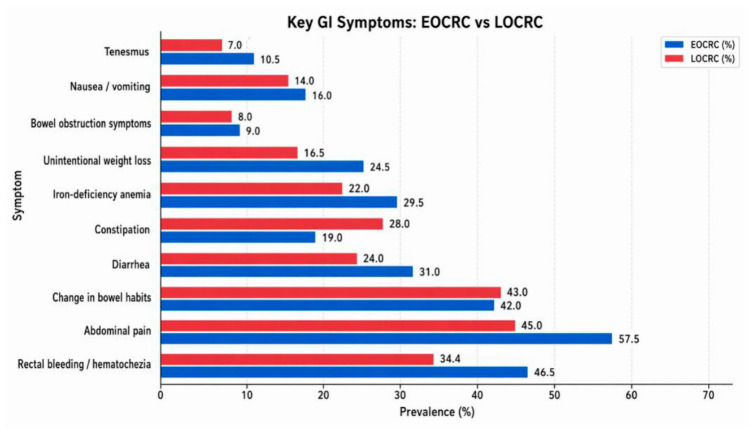
Gastrointestinal symptom profiles in EOCRC versus LOCRC.

Clinical presentation in the 6 months preceding CRC diagnosis differed between EOCRC and LOCRC. After Bonferroni correction for 10 symptom-level comparisons, with statistical significance defined as *p* < 0.005, EOCRC patients more frequently presented with rectal bleeding or hematochezia (46.5% vs. 34.4%), abdominal pain (57.5% vs. 45.0%), diarrhea (31.0% vs. 24.0%), iron-deficiency anemia (29.5% vs. 22.0%), unintentional weight loss (24.5% vs. 16.5%), and tenesmus (10.5% vs. 7.0%). In contrast, constipation was more common in LOCRC patients (28.0% vs. 19.0%). Change in bowel habits (42.0% vs. 43.0%, *p* = 0.35), bowel obstruction symptoms (9.0% vs. 8.0%, *p* = 0.09), and nausea/vomiting (16.0% vs. 14.0%, *p* = 0.01) did not meet the Bonferroni-corrected threshold for statistical significance (Table 2, Figure 1).

#### 3.1.3. Tumor Location and Location-Specific Symptom Enrichment

**Figure 2:**
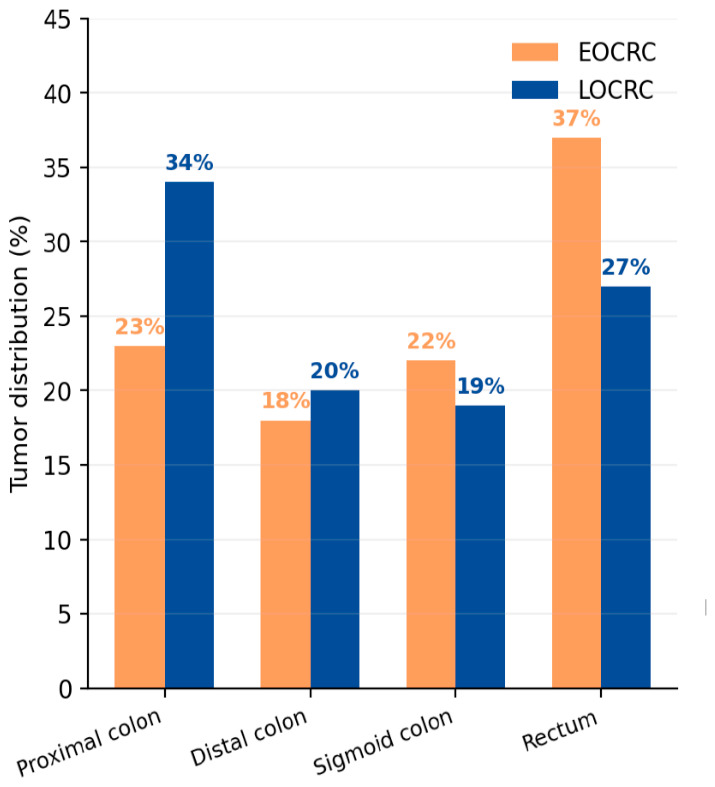
Tumor location distribution in EOCRC and LOCRC.

**Figure 3:**
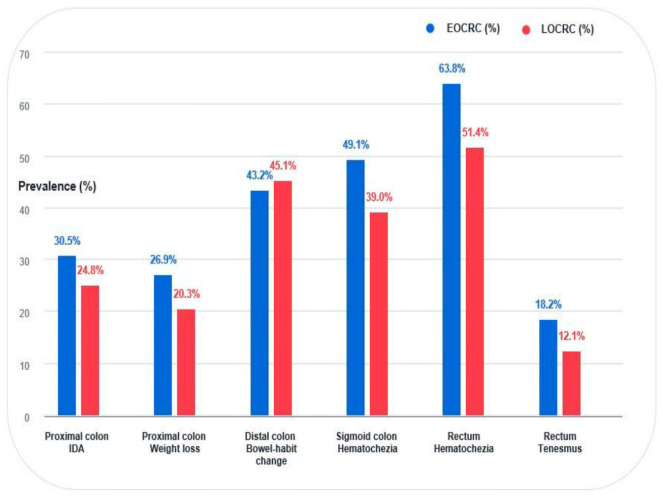
Location-specific symptom patterns in EOCRC versus LOCRC.

Tumor location also differed significantly. Rectal tumors were substantially more common in EOCRC (37.0% vs. 27.0%, *p* < 0.001), whereas proximal colon tumors predominated in LOCRC (34.0% vs. 23.0%, *p* < 0.001). Sigmoid tumors were slightly more frequent in EOCRC (22.0% vs. 19.0%, *p* = 0.001), while distal colon tumors were slightly more frequent in LOCRC (20.0% vs. 18.0%, *p* = 0.02) (Table 3, Figure 2). Within tumor-location subgroups, EOCRC was associated with more iron-deficiency anemia and weight loss in proximal colon tumors, and more rectal bleeding and tenesmus in sigmoid and rectal tumors. Overall, EOCRC showed greater symptom enrichment across several tumor-location subgroups compared with LOCRC (Table 3, Figure 3).

#### 3.1.4. Direct Multivariable Comparison of Metabolic Features Between EOCRC and LOCRC

In direct multivariable comparison, obesity-related metabolic abnormalities were more strongly associated with EOCRC. Obesity (aOR 1.38, 95% CI 1.26–1.52), metabolic syndrome (aOR 1.41, 95% CI 1.23–1.56), and MASLD (aOR 1.22, 95% CI 1.06–1.40) were more common in EOCRC, while diabetes mellitus did not differ significantly between groups (aOR 0.95, 95% CI 0.85–1.06).

### 3.2. EOCRC and LOCRC Comparison with Matched Cancer-Free Controls

#### 3.2.1. Prediagnostic Metabolic and Laboratory Profile of EOCRC and LOCRC Compared with Matched Controls

**Figure 4:**
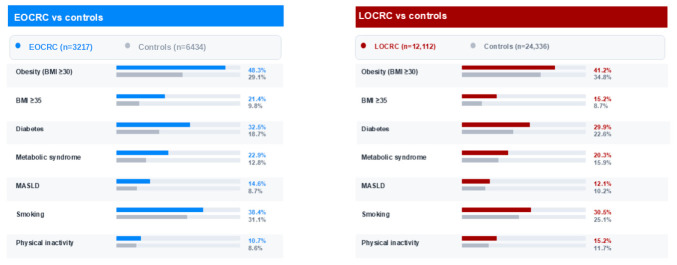
Prediagnostic clinical and metabolic characteristics of EOCRC and LOCRC compared with matched controls.

**Figure 5:**
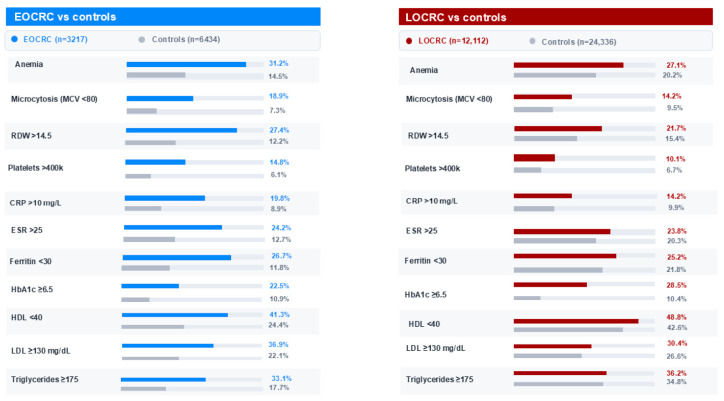
Prediagnostic hematologic, metabolic, and inflammatory laboratory abnormalities in EOCRC and LOCRC compared with matched controls.

In the separate control-based matched analyses, 3217 patients with EOCRC and 12,112 patients with LOCRC were compared with 6434 and 24,336 matched cancer-free controls, respectively. These denominators reflect age-specific case–control matching and are analytically distinct from the direct EOCRC-versus-LOCRC matched comparison. After Bonferroni correction, with statistical significance defined as *p* < 0.0014, both EOCRC and LOCRC were associated with a higher burden of prediagnostic metabolic, hematologic, inflammatory, glycemic, and lipid abnormalities compared with their respective controls. In EOCRC, obesity, diabetes mellitus, metabolic syndrome, and MASLD remained significantly more frequent than in controls. In LOCRC, obesity and metabolic syndrome remained significant, whereas diabetes mellitus, MASLD, smoking, and physical inactivity did not meet the Bonferroni-corrected threshold. Hematologic abnormalities suggestive of iron deficiency, inflammatory markers, dyslipidemia, elevated HbA1c, and thrombocytosis were more frequent in both EOCRC and LOCRC compared with matched controls (Table 4, Figure 4 and Figure 5).

#### 3.2.2. Variables Independently Associated with EOCRC and LOCRC

**Figure 6:**
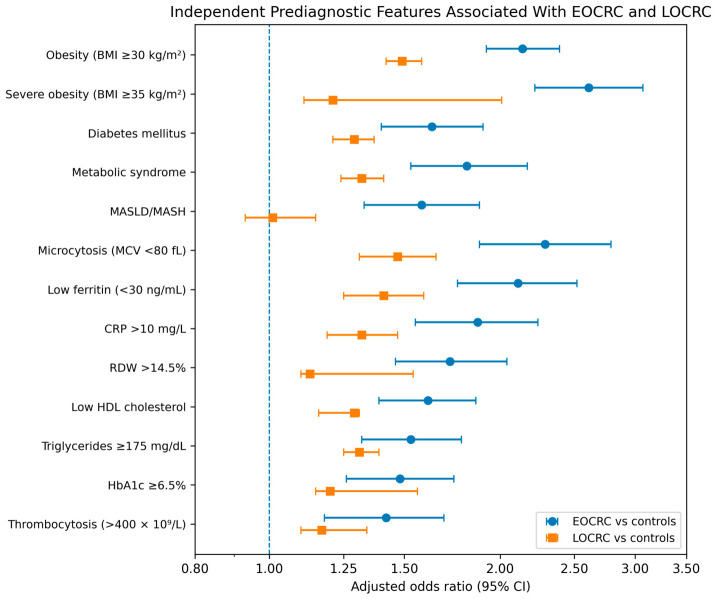
Forest plot of adjusted odds ratios and 95% confidence intervals for independent prediagnostic clinical and laboratory features associated with EOCRC and LOCRC compared with matched controls. Values are derived from the multivariable conditional logistic regression results shown in Table 5. The vertical dashed line represents the null value of an adjusted odds ratio of 1.0; estimates to the right indicate higher odds of the feature compared with controls.

In multivariable conditional logistic regression, EOCRC and LOCRC shared several variables independently associated with CRC, including obesity, metabolic syndrome, microcytosis, low ferritin, elevated CRP, low HDL cholesterol, hypertriglyceridemia, elevated HbA1c, and thrombocytosis; however, the magnitude of association was consistently greater for EOCRC. In EOCRC, the strongest independent associations were severe obesity (aOR 2.61, 95% CI 2.22–3.07), microcytosis (aOR 2.29, 95% CI 1.88–2.79), low ferritin (aOR 2.11, 95% CI 1.76–2.52), elevated CRP (aOR 1.87, 95% CI 1.55–2.24), and metabolic syndrome (aOR 1.81, 95% CI 1.53–2.17). In contrast, the strongest LOCRC associations were of lower magnitude, including obesity, microcytosis, low ferritin, elevated CRP, metabolic syndrome, and hypertriglyceridemia. MASLD was independently associated with EOCRC (aOR 1.58, 95% CI 1.33–1.88), but not LOCRC (aOR 1.01, 95% CI 0.93–1.15) (Table 5, Figure 6).

## 4. Discussion

In this multicenter matched case–control study, EOCRC was associated with a more pronounced metabolic, inflammatory, and laboratory profile compared with LOCRC. Variables associated with EOCRC generally demonstrated stronger effect sizes than those observed for LOCRC. The principal contribution of this study is confirmatory and integrative: it evaluates symptoms, tumor location, metabolic disease, and routine laboratory biomarkers within a unified EHR-based framework and directly contrasts early-onset and late-onset disease. The findings support EOCRC as a distinct clinical–metabolic phenotype, but they should not be interpreted as establishing causality or a validated screening model [23].

The observed associations must be interpreted in the context of possible reverse causation. Several abnormalities measured during the 24 months before diagnosis, including low ferritin, microcytosis, elevated CRP, thrombocytosis, weight loss, and glycemic or lipid perturbations, may reflect occult malignancy, tumor-associated inflammation, or cancer-related metabolic remodeling rather than antecedent causal exposures. Accordingly, the term prediagnostic is used throughout this manuscript to describe timing of measurement rather than biological temporality. Prospective studies with serial measurements, lagged exposure windows, and exclusion of biomarkers measured close to diagnosis will be necessary to clarify whether these features precede tumor initiation or represent early manifestations of undiagnosed CRC.

A notable strength of our study is the direct comparison between EOCRC and LOCRC, which revealed important differences in patient characteristics, symptom patterns, tumor distribution, and multivariable associations. EOCRC patients more frequently presented with rectal bleeding, abdominal pain, diarrhea, iron-deficiency anemia, weight loss, and tenesmus, whereas constipation was more common in LOCRC. In parallel, EOCRC demonstrated a distal and rectal-predominant distribution, while LOCRC was more often proximal. Within location-specific analyses, EOCRC remained associated with a greater symptomatic burden, including more iron-deficiency anemia and weight loss in proximal tumors and more rectal bleeding and tenesmus in rectal tumors. On direct multivariable comparison, obesity, metabolic syndrome, and MASLD were more strongly associated with EOCRC. Collectively, these findings suggest that EOCRC differs from LOCRC not only by age at diagnosis but also by clinical presentation, anatomic distribution, and associated metabolic profile.

Obesity showed one of the strongest independent associations with EOCRC, with adjusted odds ratios of 2.14 for BMI ≥ 30 kg/m^2^ and 2.61 for BMI ≥ 35 kg/m^2^, compared with more attenuated associations in LOCRC. These effect sizes appear larger than those reported in prior reviews and meta-analyses [12,24,25], which may reflect differences in study design, population characteristics, exposure ascertainment, and the prediagnostic assessment window. Similarly, metabolic syndrome, diabetes, and MASLD were more strongly associated with EOCRC than LOCRC. These findings are consistent with prior studies linking metabolic dysfunction to colorectal carcinogenesis through pathways involving insulin resistance, hyperinsulinemia, chronic inflammation, and IGF-1-mediated tumor promotion [9,10,26,27]. However, given the retrospective observational design, these associations should be interpreted as prediagnostic correlates rather than evidence of causality.

Mendelian randomization analyses from the GECCO/CORECT/CCFR consortium have reported evidence supporting causal associations between body fat percentage, waist circumference, fasting insulin, and EOCRC risk [28]. In this context, the stronger associations observed in our study between metabolic abnormalities and EOCRC provide complementary real-world evidence supporting the relevance of metabolic dysfunction in younger-onset disease. The association between MASLD and EOCRC is particularly noteworthy, as this relationship remains insufficiently examined in EOCRC-specific studies. Prior evidence that bariatric surgery is associated with reduced future CRC incidence provides additional indirect support for the potential role of metabolic health in colorectal carcinogenesis [29]. Nevertheless, the present findings cannot determine whether metabolic abnormalities represent causal risk factors, shared correlates, or markers of early disease biology.

A distinguishing feature of our study is the systematic evaluation of routine laboratory biomarkers as prediagnostic features associated with EOCRC. Microcytosis, low ferritin, elevated CRP, increased RDW, and thrombocytosis formed a constellation of hematologic and inflammatory abnormalities that were more strongly associated with EOCRC than LOCRC. Fritz et al. identified iron-deficiency anemia as one of four red-flag signs predictive of EOCRC, with a 6.5-fold increased risk when three or more signs co-occur [15]. Our results extend this observation by demonstrating that individual laboratory components of iron deficiency, including microcytosis and low ferritin, were independently associated with EOCRC after adjustment for metabolic and clinical covariates.

Elevated RDW, an established prognostic marker in CRC [30], also emerged as a prediagnostic marker more strongly associated with EOCRC. This may reflect subclinical hematologic derangement before diagnosis, although whether these abnormalities precede tumor development or reflect early cancer-related effects cannot be determined from the present study. Similarly, the stronger association between elevated CRP and EOCRC may reflect a more prominent inflammatory component in younger-onset disease, potentially interacting with concurrent metabolic dysfunction [4,31]. Dyslipidemia, including low HDL cholesterol and hypertriglyceridemia, was also independently associated with EOCRC, consistent with prior dose–response evidence linking elevated triglycerides with CRC risk [17]. Together, these findings suggest that routinely available laboratory abnormalities may help identify younger adults with a higher-risk clinical profile, but prospective validation is needed before clinical implementation.

Sex- and race/ethnicity-specific patterns remain important areas for further study. EOCRC incidence and outcomes differ by sex, race, ethnicity, and structural access to care, and these factors may modify the relationship between metabolic dysfunction, laboratory abnormalities, diagnostic delay, and tumor location [32]. Although our matching strategy accounted for sex, race, ethnicity, prior colonoscopy, and healthcare utilization, the present analysis did not formally test interaction terms or provide fully stratified multivariable models. Future analyses should evaluate sex-specific and race/ethnicity-stratified effect estimates and formally test heterogeneity between EOCRC and LOCRC associations.

This study has several limitations. The retrospective design and reliance on EHR-derived data from the TriNetX network may introduce coding inaccuracies and selection bias, as patients with more frequent healthcare encounters may have more thoroughly documented diagnoses and laboratory values. Several exposures, including MASLD, smoking, alcohol use, and physical inactivity, are particularly dependent on documentation and coding practices and may be underreported or differentially captured across health systems. Although rigorous 1:2 matching was performed to balance measured covariates, residual confounding from unmeasured variables cannot be excluded. Incomplete laboratory capture across participating healthcare systems may contribute to information bias; therefore, multiple imputation was applied to address missing laboratory values, but imputation cannot fully eliminate bias from nonrandom testing patterns. The temporal relationship between laboratory abnormalities and cancer diagnosis cannot establish causality; some laboratory derangements observed in the two-year prediagnostic window may reflect early tumor effects rather than antecedent risk factors. We did not conduct alternative exposure-window sensitivity analyses, formal interaction testing for heterogeneity between EOCRC and LOCRC effect estimates, sex-specific analyses, race/ethnicity-stratified multivariable analyses, or stage-adjusted analyses. Tumor stage at diagnosis was unavailable and could partly explain inflammatory, hematologic, or metabolic abnormalities if EOCRC patients presented with more advanced disease. The absence of tumor molecular data, including microsatellite instability, KRAS, NRAS, BRAF, APC, TP53, and CpG island methylation status, limits biological interpretation and precludes linkage of metabolic phenotypes to molecular CRC subtypes. External validation in independent resources such as UK Biobank, All of Us, SEER-Medicare, or other real-world datasets was not performed; therefore, reproducibility beyond the TriNetX environment remains unknown. Finally, no prediction model was developed, and the study did not evaluate discrimination, calibration, decision-curve benefit, or clinical utility. Therefore, the present findings should be viewed as hypothesis-generating associations that may inform future model development rather than as evidence supporting immediate EHR-based screening implementation.

Future work should move beyond single-database association analyses toward prospective, externally validated, multimodal risk models. Such models should follow established model-development frameworks with prespecified training and validation cohorts, ROC/AUC estimation, calibration assessment, decision-curve analysis, and evaluation of clinical net benefit [33]. For EOCRC specifically, future risk tools may need to integrate routine laboratory data with tumor location, family history, medication exposures, lifestyle factors, germline and somatic molecular features, microbiome signatures, liquid biopsy approaches such as circulating cell-free DNA methylation, and, where clinically appropriate, imaging biomarkers [34]. These approaches may offer greater discriminatory power than routine laboratory markers alone and could help determine whether risk-enriched evaluation before age 45 is clinically justified.

## 5. Conclusions

In this large multicenter matched case–control study, EOCRC was associated with a more pronounced metabolic, inflammatory, and hematologic profile than LOCRC, with stronger independent associations for obesity, metabolic syndrome, iron-deficiency-related laboratory abnormalities, and inflammatory markers. EOCRC also demonstrated a distinct clinical phenotype characterized by greater symptomatic burden and a distal- and rectal-predominant tumor distribution, whereas LOCRC was more often proximally located. These findings support EOCRC as a distinct clinical and metabolic phenotype but should be interpreted as prediagnostic associations rather than validated predictors or causal risk factors. Future prospective studies with external validation, stage and molecular data, formal heterogeneity testing, alternative exposure windows, and predictive-performance assessment are warranted to evaluate risk-based approaches for earlier identification of younger adults at increased risk for CRC.

## Figures and Tables

**Table 1:** Baseline Characteristics of Matched Patients With Early-Onset and Late-Onset Colorectal Cancer.

Characteristic	EOCRC (N = 2584)	LOCRC (N = 5168)	*p* Value	SMD
**Demographics**				
Age, years	42.8 ± 5.9	63.4 ± 7.1	<0.001	3.151
Female	1214 (47.0)	2433 (47.1%)	0.94	0.002
Male	1370 (53.0)	2735 (52.9%)	0.45	0.02
White	1509 (58.4)	3269 (63.3%)	<0.001	0.100
Black	430 (16.6%)	668 (12.9%)	<0.001	0.105
Hispanic	413 (16.0)	694 (13.4%)	0.001	0.076
Asian	129 (5.0)	331 (6.4%)	0.004	0.061
**BMI**				
<25 kg/m^2^	569 (22.0%)	1395 (27.0%)	<0.001	0.115
25–29.9 kg/m^2^	775 (30.0%)	1705 (33.0%)	0.002	0.064
30–34.9 kg/m^2^	718 (27.8%)	1292 (25.0%)	0.003	0.063
≥35 kg/m^2^	522 (20.2%)	775 (15.0%)	<0.001	0.137
**Comorbidities**				
Diabetes mellitus	697 (27.0%)	1447 (28.0%)	0.28	0.022
Hypertension	646 (25.0%)	2067 (40.0%)	<0.001	0.322
Smoking	714 (27.6%)	1175 (22.7%)	<0.001	0.112
Obesity (BMI ≥ 30 kg/m^2^)	1240 (48.0%)	2067 (40.0%)	<0.001	0.161
Metabolic syndrome	555 (21.5%)	853 (16.5%)	<0.001	0.127
MASLD/MASH	353 (13.7%)	594 (11.5%)	0.002	0.066

Abbreviations: EOCRC, early-onset colorectal cancer; LOCRC, late-onset colorectal cancer; SMD, standardized mean difference.

**Table 2:** Gastrointestinal Symptom Profiles in Matched Patients With EOCRC and LOCRC.

Symptom	EOCRC (N = 2584)	LOCRC (N = 5168)	*p* Value	SMD
Rectal bleeding/hematochezia	1202 (46.5%)	1776 (34.4%)	<0.001	0.249
Abdominal pain	1487 (57.5%)	2326 (45.0%)	<0.001	0.252
Change in bowel habits	1085 (42.0%)	2222 (43.0%)	0.35	0.020
Diarrhea	801 (31.0%)	1240 (24.0%)	<0.001	0.158
Constipation	491 (19.0%)	1447 (28.0%)	<0.001	0.211
Iron-deficiency anemia	763 (29.5%)	1137 (22.0%)	<0.001	0.173
Unintentional weight loss	633 (24.5%)	853 (16.5%)	<0.001	0.200
Bowel obstruction symptoms	233 (9.0%)	413 (8.0%)	0.09	0.036
Nausea/vomiting	413 (16.0%)	724 (14.0%)	0.01	0.057
Tenesmus	271 (10.5%)	362 (7.0%)	<0.001	0.123

EOCRC, early-onset colorectal cancer; LOCRC, late-onset colorectal cancer; SMD, standardized mean difference.

**Table 3:** Tumor Location and Location-Specific Symptom Patterns in EOCRC Versus LOCRC.

Tumor Location	EOCRC (N = 2584)	LOCRC (N = 5168)	*p* Value	SMD
Proximal colon	594 (23.0%)	1757 (34.0%)	<0.001	0.243
Distal colon	465 (18.0%)	1034 (20.0%)	0.02	0.051
Sigmoid colon	568 (22.0%)	982 (19.0%)	0.001	0.074
Rectum	956 (37.0%)	1395 (27.0%)	<0.001	0.217
**Symptom specific location**				
Proximal colon: Iron-deficiency anemia	30.5%	24.8%	<0.001	0.128
Proximal colon: Weight loss	26.9%	20.3%	<0.001	0.157
Distal colon: Change in bowel habits	43.2%	45.1%	0.38	0.038
Sigmoid colon: Rectal bleeding	49.1%	39.0%	<0.001	0.205
Rectum: Rectal bleeding	63.8%	51.4%	<0.001	0.253
Rectum: Tenesmus	18.2%	12.1%	<0.001	0.174

EOCRC, early-onset colorectal cancer; LOCRC, late-onset colorectal cancer; SMD, standardized mean difference.

**Table 4:** Prediagnostic Metabolic and Laboratory Characteristics of Patients with EOCRC and LOCRC Compared with Matched Cancer-Free Controls.

Variables	EOCRC (n = 3217)	Controls (n = 6434)	*p*-Value	LOCRC (n = 12,112)	Controls (n = 24,336)	*p*-Value
Obesity (BMI ≥ 30)	1554 (48.3)	1873 (29.1)	<0.001	4990 (41.2)	8469 (34.8)	<0.001
Severe obesity (BMI ≥ 35)	688 (21.4)	631 (9.8)	<0.001	1841 (15.2)	2117 (8.7)	<0.001
Diabetes	1046 (32.5)	1203 (18.7)	<0.001	3622 (29.9)	5501 (22.6)	0.005
Metabolic syndrome	737 (22.9)	824 (12.8)	<0.001	2459 (20.3)	3869 (15.9)	<0.001
MASLD/MASH	470 (14.6)	560 (8.7)	<0.001	1466 (12.1)	2482 (10.2)	0.008
Smoking	1235 (38.4)	2001 (31.1)	0.005	3694 (30.5)	6110 (25.1)	0.008
Physical inactivity	344 (10.7)	553 (8.6)	0.004	1841 (15.2)	2847 (11.7)	0.002
Anemia	1004 (31.2)	933 (14.5)	<0.001	3282 (27.1)	4916 (20.2)	<0.001
Microcytosis (MCV < 80)	608 (18.9)	470 (7.3)	<0.001	1720 (14.2)	2312 (9.5)	<0.001
RDW > 14.5	881 (27.4)	785 (12.2)	<0.001	2628 (21.7)	3749 (15.4)	<0.001
Platelets > 400 k	476 (14.8)	392 (6.1)	<0.001	1223 (10.1)	1631 (6.7)	<0.001
HbA1c ≥ 6.5	724 (22.5)	701 (10.9)	<0.001	3452 (28.5)	2531 (10.4)	<0.001
HDL < 40	1329 (41.3)	1570 (24.4)	<0.001	5911 (48.8)	10,369 (42.6)	<0.001
LDL ≥ 130 mg/dL	1187 (36.9)	1422 (22.1)	<0.001	3682 (30.4)	6474 (26.6)	<0.001
Triglycerides ≥ 175	1065 (33.1)	1139 (17.7)	<0.001	4385 (36.2)	8469 (34.8)	<0.001
CRP > 10 mg/L	637 (19.8)	573 (8.9)	<0.001	1720 (14.2)	2410 (9.9)	<0.001
ESR > 25	779 (24.2)	817 (12.7)	<0.001	2882 (23.8)	4941 (20.3)	<0.001
Ferritin < 30	859 (26.7)	759 (11.8)	<0.001	3052 (25.2)	5304 (21.8)	<0.001

BMI, body mass index; CRP, C-reactive protein; EOCRC, early-onset colorectal cancer; ESR, erythrocyte sedimentation rate; HbA1c, hemoglobin A1c; HDL, high-density lipoprotein; LDL, low-density lipoprotein; LOCRC, late-onset colorectal cancer; MCV, mean corpuscular volume; RDW, red cell distribution width.

**Table 5:** Multivariable Conditional Logistic Regression Analysis of Independent Factors Associated With EOCRC and LOCRC.

Variables	EOCRC vs. Controls		LOCRC vs. Controls	
	aOR (95% CI)	*p* Value	aOR (95% CI)	*p* Value
**Clinical variables**				
Obesity (BMI ≥ 30 kg/m^2^)	2.14 (1.92–2.39)	<0.01	1.49 (1.42–1.58)	<0.01
Severe obesity (BMI ≥ 35 kg/m^2^)	2.61 (2.22–3.07)	<0.001	1.21 (1.11–2.01)	<0.01
Diabetes mellitus	1.63 (1.40–1.90)	<0.01	1.29 (1.21–1.37)	<0.01
Metabolic syndrome	1.81 (1.53–2.17)	<0.01	1.32 (1.24–1.41)	<0.01
MASLD/MASH	1.58 (1.33–1.88)	<0.01	1.01 (0.93–1.15)	0.12
**Laboratory variables**				
Microcytosis (MCV < 80 fL)	2.29 (1.88–2.79)	<0.01	1.47 (1.31–1.65)	<0.01
Low ferritin (<30 ng/mL)	2.11 (1.76–2.52)	<0.01	1.41 (1.25–1.59)	<0.01
C-reactive protein > 10 mg/L	1.87 (1.55–2.24)	<0.01	1.32 (1.19–1.47)	<0.01
Red cell distribution width > 14.5%	1.72 (1.46–2.04)	<0.01	1.13 (1.10–1.54)	<0.01
Low HDL cholesterol	1.61 (1.39–1.86)	<0.01	1.29 (1.16–1.31)	<0.01
Triglycerides ≥ 175 mg/dL	1.53 (1.32–1.78)	<0.01	1.31 (1.25–1.39)	<0.01
Hemoglobin A1c ≥ 6.5%	1.48 (1.26–1.74)	<0.01	1.20 (1.15–1.56)	<0.01
Thrombocytosis (>400 × 10^9^/L)	1.42 (1.18–1.69)	<0.01	1.17 (1.10–1.34)	<0.01

Abbreviations: aOR, adjusted odds ratio; BMI, body mass index; EOCRC, early-onset colorectal cancer; LOCRC, late-onset colorectal cancer; MCV, mean corpuscular volume.

## Data Availability

The data supporting the findings of this study are available from the TriNetX U.S. Network. Restrictions apply to the availability of these data, which were used under license for the current study and are not publicly available. Aggregated data may be available from the corresponding author upon reasonable request and subject to TriNetX data-use agreements.

## Supplementary Materials

The following supporting information can be downloaded at https://www.mdpi.com/article/10.3390/cancers18132152/s1. Supplementary Table S1: STROBE checklist for case–control studies. Supplementary Table S2: Codes utilized in our study used to define our study cohorts and variables.
