# Supplementary material for: Metabolic and Laboratory Biomarkers in Early-Onset Versus Late-Onset Colorectal Cancer: A Case–Control Study
Source: Cancers (Basel). 2026 Jul 3;18(13):2152. doi: 10.3390/cancers18132152 (PMC13359632; doi:10.3390/cancers18132152)
Supplement: Supplementary file 1 [file cancers-18-02152-s001.zip › cancers-4387992-supplementary.pdf]

Supplementary Table S1. Completed STROBE Checklist for This Study				
**Section/topic**	**Item**	**STROBE recommendation**	**Manuscript location**	**Checklist comments**
Title and abstract	1a	Indicate the study's design with a commonly used term in the title or the abstract.	Page 1, title and Methods in abstract	Title states 'matched case-control study'; abstract Methods states 'multicenter matched case-control study'.
Title and abstract	1b	Provide in the abstract an informative and balanced summary of what was done and what was found.	Page 1, abstract	Abstract includes background, methods, key results with cohort sizes and adjusted odds ratios, and a conclusion.
Introduction - Background/rationale	2	Explain the scientific background and rationale for the investigation being reported.	Page 2, Introduction	Rationale describes rising EOCRC incidence, metabolic dysfunction, inflammation, laboratory abnormalities, and the gap in EOCRC versus LOCRC comparisons.
Introduction - Objectives	3	State specific objectives, including any prespecified hypotheses.	Page 2, final Introduction paragraph	Objective stated: identify prediagnostic clinical/laboratory features associated with EOCRC and determine whether factors differ from LOCRC.
Methods - Study design	4	Present key elements of study design early in the paper.	Page 3, section 2.1	Design is stated as a multicenter matched case-control study using TriNetX U.S. Network.
Methods - Setting	5	Describe the setting, locations, and relevant dates, including periods of recruitment, exposure, follow-up, and data collection.	Pages 3-4, sections 2.1-2.3	Setting is TriNetX U.S. Network with 67 health care organizations. Dates are CRC diagnosis from January 2010 to December 2023; symptoms were assessed 6 months before index; clinical/laboratory variables 24 months before index.
Methods - Participants	6a	Give the eligibility criteria, and the sources and methods of case ascertainment and control selection. Give the rationale for the choice of cases and controls.	Pages 3-4, section 2.2	Eligibility, case definitions, exclusions, index date, age-defined EOCRC/LOCRC cohorts, and cancer-free controls are described. Consider adding one sentence explaining the rationale for choosing cancer-free controls as the reference group if the journal asks for strict STROBE wording.
Methods - Participants	6b	For matched studies, give matching criteria and the number of controls per case.	Pages 4-5, sections 2.2 and 2.4	Case-control analyses used up to two controls per case. Direct EOCRC-versus-LOCRC matching and control-based matching variables are listed.
Methods - Variables	7	Clearly define all outcomes, exposures, predictors, potential confounders, and effect modifiers. Give diagnostic criteria, if applicable.	Pages 3-5, sections 2.2-2.4	EOCRC/LOCRC, symptoms, tumor location comparisons, metabolic/laboratory variables, thresholds, exclusions, matching variables, and control groups are described. Diagnostic code lists are referred to as Supplementary Table 1.
Methods - Data sources/measurement	8	For each variable of interest, give sources of data and details of methods of assessment (measurement). Describe comparability of assessment methods if there is more than one group.	Pages 3-4, sections 2.1-2.3	Data sources include ICD-9/ICD-10-CM, ICD-10-PCS, CPT, LOINC, RxNorm, Veterans Affairs medication codes, and TriNetX clinical observation codes. Variables were ascertained in the same pre-index windows across groups.
Methods - Study size	9	Explain how the study size was arrived at.	Pages 3-7	The study appears to include all eligible patients in TriNetX meeting criteria, and final analytic sample sizes are reported. Consider adding: 'No formal sample-size calculation was performed; all eligible patients meeting criteria were included.'
Methods - Quantitative variables	10	Explain how quantitative variables were handled in the analyses. If applicable, describe which groupings were chosen and why.	Pages 4-5, sections 2.3-2.4	Laboratory and BMI thresholds are described as clinically relevant and predefined; most recent pre-index laboratory value was used.
Methods - Statistical methods	11a	Describe all statistical methods, including those used to control for confounding.	Page 5, section 2.4	Matching, conditional logistic regression, adjusted odds ratios with 95% confidence intervals, multiple imputation, Firth penalized regression, Bonferroni correction, and two-sided testing are described.
Methods - Statistical methods	12b	Describe any methods used to examine subgroups and interactions.	Pages 4, 6-7	Location-specific symptom analyses and separate EOCRC/LOCRC control-based analyses are reported. The manuscript does not describe formal interaction testing; if none was done, no change is needed, or add 'No formal interaction testing was performed.'
Methods - Statistical methods	12c	Explain how missing data were addressed.	Page 5, section 2.4	Missing BMI and laboratory data were handled using multiple imputation.
Methods - Statistical methods	12d	If applicable, explain how matching of cases and controls was addressed.	Pages 4-5, sections 2.2 and 2.4	Matching variables and controls per case are reported; conditional logistic regression was used to account for the matched design.
Results - Participants	13a	Report numbers of individuals at each stage of study, such as numbers potentially eligible, examined for eligibility, confirmed eligible, included in the study, completing follow-up, and analysed.	Pages 5-7, Results	Final analytic sample sizes are reported for direct and control-based analyses. Counts at each eligibility/exclusion/matching stage are not shown in the supplied manuscript.

Supplementary Table S1. Completed STROBE Checklist for This Study				
**Section/topic**	**Item**	**STROBE recommendation**	**Manuscript location**	**Checklist comments**
Results - Participants	13b	Give reasons for non-participation at each stage.	Pages 3-4 and Results	This is an EHR database study, so participant non-participation is not applicable in the traditional sense. Exclusion criteria are described, but exclusion counts by reason are not reported.
Results - Descriptive data	14	Give characteristics of study participants and information on exposures and potential confounders.	Pages 5-7; Tables 1 and 4 referenced	Baseline characteristics, exposures, and potential confounders are summarized in the Results text and referenced tables.
Results - Outcome data	15	Report numbers in each exposure category, or summary measures of exposure.	Pages 5-7; Tables 1-5 referenced	Exposure/category summaries and adjusted estimates are reported in Results and referenced tables. The current supplied DOCX does not include the actual tables, so verify that the submission package includes the complete tables.
Results - Main results	16a	Give unadjusted estimates and, if applicable, confounder-adjusted estimates and their precision. Make clear which confounders were adjusted for and why they were included.	Pages 6-7; Table 5 referenced	Adjusted odds ratios and 95% confidence intervals are reported. The manuscript also states unadjusted p-values are in tables. Consider making the exact multivariable adjustment set explicit in the table footnotes.
Results - Main results	16b	Report category boundaries when continuous variables were categorized.	Pages 4-5, section 2.3	BMI and laboratory category thresholds are described; verify table footnotes repeat thresholds for reader clarity.
Results - Other analyses	17	Report other analyses done, such as analyses of subgroups and interactions, and sensitivity analyses.	Pages 6-7	Location-specific symptom enrichment and direct EOCRC-versus-LOCRC analyses are reported. No sensitivity analyses are reported.
Discussion - Key results	18	Summarise key results with reference to study objectives.	Pages 7-11, Discussion	Key findings are summarized in relation to EOCRC versus LOCRC phenotype and prediagnostic metabolic/laboratory profile.
Discussion - Limitations	19	Discuss limitations of the study, taking into account sources of potential bias or imprecision. Discuss both direction and magnitude of any potential bias.	Page 10, limitations paragraph	Limitations include retrospective design, EHR coding, selection bias, residual confounding, missing labs, temporal ambiguity, absent molecular data, unavailable lifestyle variables, and generalizability. Direction/magnitude of bias is discussed qualitatively but not quantitatively.
Discussion - Interpretation	20	Give a cautious overall interpretation of results considering objectives, limitations, multiplicity of analyses, results from similar studies, and other relevant evidence.	Pages 8-11	Interpretation is cautious, acknowledges observational design and multiplicity, and contextualizes results with prior literature.
Discussion - Generalisability	21	Discuss the generalisability/external validity of the study results.	Page 10, limitations paragraph	The manuscript states findings are from a U.S. healthcare network and may not be generalizable to other populations.

Supplementary Table S2: Codes utilized in our study used to define our study cohorts and variables	
**Study variable**	**Codes**
CRC diagnosis	C18.0; C19; C20
Prior malignancy	C00-C97
Inflammatory bowel disease	K50; K51
Hereditary or familial CRC risk	Z15.09; Z80.0; Z84.81
Prior colectomy	44140, 44141, 44143, 44144, 44145, 44146, 44147, 44150, 44151, 44155, 44156, 44157, 44158, 44160; 44204, 44205, 44206, 44207, 44208, 44210, 44211, 44212, Z90.49
BMI	Z68.20-Z68.24; Z68.25-Z68.29; Z68.30-Z68.34; Z68.35-Z68.45
Obesity	E66; Z68.30-Z68.45<br>E66.01; E66; Z68.35-Z68.45
Diabetes mellitus	E08; E09; E10; E11; E13
Hypertension	I10-I15
Smoking / tobacco exposure	F17; Z72.0; Z87.891; Z77.22
Metabolic syndrome	E88.81
MASLD/MASH / NAFLD/NASH	K76.0; K75.81
Physical inactivity	Z72.3
Rectal bleeding / hematochezia	K62.5; K92.1
Abdominal pain	R10
Change in bowel habits	R19.4
Diarrhea	R19.7
Constipation	K59.0
Iron-deficiency anemia	D50, 85025, 85027, 85018, 85014; 82728
Unintentional weight loss	R63.4
Bowel obstruction symptoms	K56; R14
Nausea / vomiting	R11
Tenesmus	R19.8
Proximal colon	C18
Distal colon	C18.5; C18.6
Sigmoid colon	C18.7
Rectosigmoid junction	C19
Rectum	C20
Overlapping/unspecified colon	C18.8; C18.9
Anemia	D50; D64.9, 85025, 85027, 85018, 85014
Microcytosis (MCV <80 fL)	R71.8; D50, 85025, 85027
RDW >14.5%	R71.8, 85025, 85027
Platelets >400k / thrombocytosis	D75.83; D47.3, 85025, 85027, 85049
HbA1c >=6.5%	R73.09; E11, 83036, 83037
HDL cholesterol <40 mg/dL	E78.6, 80061, 83718
LDL cholesterol >=130 mg/dL	E78.0; E78.2; E78.5, 80061, 83721
Triglycerides >=175 mg/dL	E78.1; E78.2, 80061, 84478
C-reactive protein >10 mg/L	R79.82, 86140, 86141
ESR >25 mm/hr	R70.0, 85651, 85652
Ferritin <30 ng/mL	E61.1; D50; R79.0, 82728
